# Design of a Human Evaluator Model for the Ride Comfort of Vehicle on a Speed Bump Using a Neural Artistic Style Extraction

**DOI:** 10.3390/s19245407

**Published:** 2019-12-08

**Authors:** Donggyun Kim, MyeonGyu Jeong, ByungGuk Bae, Changsun Ahn

**Affiliations:** 1 School of Mechanical Engineering, Pusan National University, Busan 46241, Korea; 2 Research & Development Division, Hyundai Motor Company, Hwaseong-si, Gyeonggi 18280, Korea

**Keywords:** deep neural network, neural artistic extraction, objectification, ride comfort, subjective evaluation

## Abstract

The subjective evaluation of vehicle ride comfort is costly and time-consuming but is crucial for vehicle development. To reduce the cost and time, the objectification of subjective evaluation has been widely studied, and most of the approaches use a regression model between objective metrics and subjective ratings. However, the accuracy of these approaches is highly dependent on the selection of the objective metrics. In most of the methods, it is not clear that the selected metrics are sufficiently significant or whether all significant metrics are included in the selection. This paper presents a method to build a correlation model between measurements and subjective evaluations without using predefined features or objective metrics. A numerical representation of ride comfort was extracted from raw signals based on the idea of the artistic style transfer method. The correlation model was designed based on the extracted numerical representation and subjective ratings. The model has a much better accuracy than any other correlation models in the literature. This better accuracy is contributed to not only by using a neural network, but also by the extraction of the numerical representation of ride comfort using a pre-trained neural network.

## 1. Introduction

The evaluation of the quality of goods is based on human feelings in many areas, such as works of art [1,2], consumer electronics [3,4], cars [5,6], and other products [7,8]. Subjective evaluations are as important as objective metrics for engineering products because consumers evaluate the value of products based on their feelings. However, subjective evaluations are easily affected by the experience level of the evaluator, geographical locations, and the time of evaluation. Furthermore, subjective evaluations generally take a longer time than an objective evaluation. Therefore, subjective evaluation has a lower consistency and lower repeatability than measurement-based objective evaluations. Subjective evaluations also require a finished product, so feedback from a subjective evaluation cannot be considered in the beginning phase of the product design process.

Much effort has been made to take into account subjective evaluations in earlier design phases. A mockup can be used if the subjective evaluations are based on visual and tactile aspects, such as the hands-on feel of handheld devices [9]. If the evaluations are based on a comprehensive feeling, a designer can use a correlation model between the objective and subjective evaluations with a mathematical product model that generates virtual measurements for an objective evaluation, such as vehicle ride comfort [10,11], vehicle steering feel [12,13], and art education [14].

In the automotive industry, objective evaluations of ride comfort and steering feel are inevitable and crucial during the development process. Furthermore, they cannot be substituted with any other objective evaluation metrics because customers judge cars through their feel. Subjective evaluations of a car can only be performed in the very last phase of the development process, so engineers have very little chance to modify the design parameters when the subjective evaluations become available.

Professional evaluators or special test drivers are required for subjective evaluations, which can be performed only several times per day because modification of the testing car or the testing environment takes time. Due to these constraints, several approaches have been suggested to predict the results of a subjective evaluation using values measured from a real car or a simulation model. Some of examples are linear regression. Data et al. [15] investigated a method to find the best correlation between objective metrics and subjective ratings. Rothhämel et al. [16] proposed a method to find correlations using a driving simulator and a vehicle model. Nybacka and coworkers [17,18] identified the links between objective metrics using a steering robot and the subjective evaluation of expert drivers. Gil Gómez and coworkers [19,20] found correlations between objective metrics. The other methods that have been used are nonlinear regression including a fuzzy model [21] and an artificial neural network [17,18,19,20,22]. In References [17,18,19,20], a simple neural network with two hidden layers is used. Liu et al. [22] investigated a method to find correlations between signals measured by an electromyogram and subjective evaluations. However, the methods have shown a low correlation and are effective for only limited cases because a designer selects the correlation variables between the objective measurements and the subjective evaluation, so the sufficiency and optimality of the selection are not clear. Furthermore, the generality of such correlation models is not guaranteed due to the small number of evaluation data.

Deep neural network (DNN) techniques are actively applied to the design of correlation models between objective measurements and subjective evaluations. For subjective video quality evaluations, a subjective video quality prediction model was introduced based on a DNN. Varga [23] evaluated video quality through a DNN architecture consisting of a pre-trained network, transfer learning, temporal pooling, and regression layers. In the medical field, DNN techniques are also widely used. Mahendran et al. predicted major depressive disorder using a weighted average ensemble machine learning model [24]. Weber et al. [25] calculated muscle fat infiltration using a previously developed convolution neural network. In the material field, Yao et al. used the neural network model to predict subjective tactile properties from objective test results of porous polymeric materials [26].

In the automotive field, an interesting result has been reported on regarding modeling the correlation between objective and subjective evaluations of vehicle dynamic performance using a DNN technique [27]. A method was presented to identify the relationship between the objective metrics and subjective assessments. A quite meaningful correlation model was generated and can foresee the subjective characteristics of a new vehicle based on a simulation and measurements. The correlation model was trained using 22 test drivers with 51 vehicles. The number of training sets did not seem to be sufficient compared to general DNN cases. However, the number was quite large when considering the number of test drivers and cars used for a general vehicle evaluation. For typical subjective evaluations, a few test drivers drive a few cars (a test car and few reference cars), and the process takes several days. A lack of sufficient datasets is a major difficulty in applying DNN techniques for the objectification of the subjective evaluation of cars. Even though the results were quite impressive and successful, the robustness and generality of the model are not clear due to the small training datasets.

Another weakness of this approach is that the inputs to the model are predefined objective metrics, such as the yaw gain, torque dead band, and phase time lag. The use of predefined metrics raises questions about the appropriate definitions of the metrics, the selection of the best metrics, and whether the selected metrics represent human perceptions well. 

Not all artificial intelligent techniques require a large datasets. Mordvintsev et al. [28] modified an input image such that the output from a given pre-trained neural network would be as close to the expected output as possible. This method is called Deep Dream and has been used to synthesize two images to create a new image. An image synthesis method called artistic style transfer was also developed [29], which synthesizes two images by transforming the style of one image to the other image. This technique needs only two images: one for the style and the other for the content.

In this method, the network parameters are not optimized, and transforming the style of the input image requires a recursive computational or training process. The network structure and parameters use those of a pre-trained network, such as VGG-19 [30]. VGG-19 is a pre-trained convolution neural network that was developed by the Visual Geometry Group of the University of Oxford for classification tasks. This method numerically extracts the style of an image that is recognized by human senses from a raw image without using predefined metrics, such as lines or edges.

**Figure 1:**
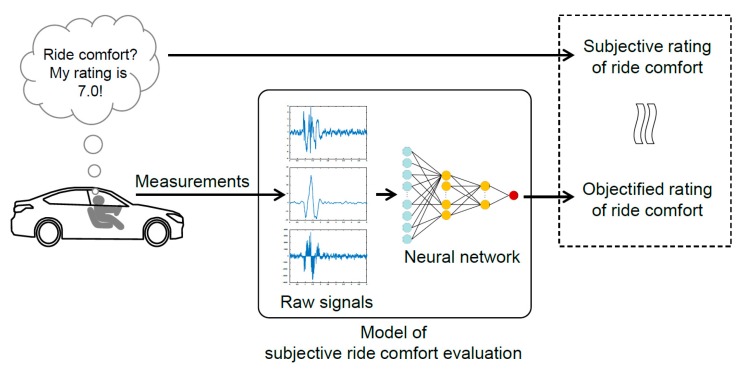
Objectification of the subjective evaluation of ride comfort.

This paper presents a method to build a correlation model between measurements and subjective evaluations without using predefined features or objective metrics, as shown in Figure 1. The key idea is that a numerical representation of ride comfort is extracted from raw signals that were measured in a test vehicle without preprocessing to define and calculate objective metrics. This method is based on the ideas of the artistic style transfer method. The proposed method was applied to the evaluation of ride comfort when a vehicle passes over a speed bump. A comparative model is proposed for the ride comfort of two vehicles to minimize the effect of using a small dataset. The input of the model is the measurements from the two vehicles, and the output is the differences in their subjective ratings of ride comfort.

The rest of the paper is organized as follows. Section 2 presents the method for the objectification of subjective evaluation, and Section 3 presents the results of the model training. Section 4 suggests possible applications of the model, and Section 5 concludes the paper.

## 2. Method

### 2.1. Data Collection

**Figure 2:**
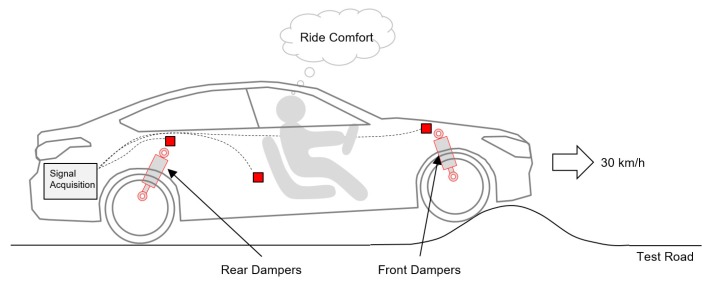
Ride comfort evaluation on a road with a speed bump.

The data used in this study were collected from ride comfort evaluations by an automotive manufacturer. In the test, a vehicle was driven at 30 km/h on a road with a speed bump, as shown in Figure 2. The test vehicle was a small sedan with several sensors. Accelerometers were installed at several positions of the vehicle to measure three-dimensional accelerations at those positions. Gyroscopes were also installed at several positions to measure three-dimensional angular rates. The front and rear dampers had three adjustable settings: hard, medium, and soft. A professional test driver evaluated the vehicle with nine combinations of dampers.

The subjective rating was reported in the form of absolute ratings defined in SAE J1441 [31], which are presented in Table 1. The ratings given by the test driver are shown in Table 2. The test driver evaluated the ride comfort in two categories: primary ride and impact comfort. Fractional expressions were used to obtain a finer resolution, which were transformed to numbers; for example, “6+” was changed to 6.33, “6+ to 7” was changed to 6.5, and “6 to 6+” was changed to 6.16.

The objective data were collected using several sensors and included the velocities, accelerations, and forces at several positions on the test vehicles. A total of 120 types of sensor signals were collected. The driver kept the speed at 30 km/h as much as possible to avoid disturbances from speed differences. The test driver drove the vehicle multiple times for each damper setting, and four sets of objective data were collected for each setting. Therefore, the total number of datasets was 36.

### 2.2. Ride Comfort Evaluation Model

**Figure 3:**
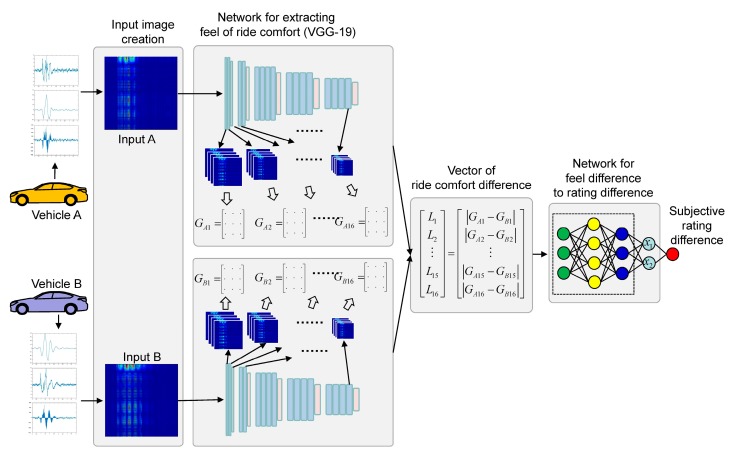
Structure of ride comfort evaluation model. VGG: Visual Geometry Group.

The basic concept of the model is as follows. First, an image was created by combining the spectrograms of measured signals, and then gram matrices were extracted from the image as numerical representations of ride comfort. An artificial neural network was then trained to find a relationship between the numerical representations and the subjective ratings. Based on this concept, we designed a model structure to predict the difference of the subjective ratings between two vehicles rather than predicting the absolute rating of ride comfort, as shown in Figure 3. A comparative model was designed to increase the number of training datasets.

Two artificial neural networks were used in the model: one for extracting a numerical representation of the ride comfort and another for the correlation model between the extracted numerical representation and the subjective ratings. Extracting ride comfort did not require any datasets for training because the extraction was performed by a pre-trained convolutional neural network (CNN). However, building the correlation model required datasets for training. The 36 datasets collected were not sufficient. We designed a comparative model to increase the number of training sets. The input was the difference between the numerical representations of ride comfort that were extracted from the measurements of two vehicles, and the output was the difference between the vehicles’ subjective ratings.

#### 2.2.1. Extraction of Ride Comfort from Measurements

**Figure 4:**
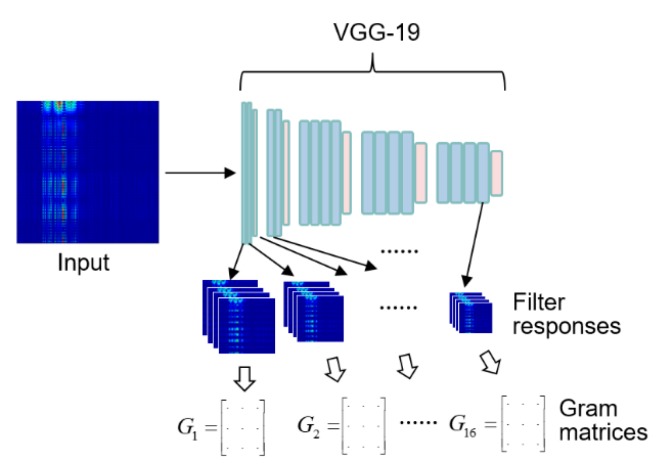
Extracting a numerical representation of ride comfort based on the ideas of the artistic style transfer algorithm. The network input is an image transformed from the measured temporal data.

Instead, the style was implicitly defined in the pre-trained network. The numerical representation of the style of an image is a set of gram matrices, [*G*_1_, *G*_2_, ···, *G*_16_], of the filter responses in the layers of VGG-19, as shown in Figure 4. Each layer produces an abstract concept of the image. In the CNN, each layer further abstracts the pixel representations of the image.

The algorithm uses the filter responses at all layers as a representation of the contents of the original image. The gram matrices of the filter responses of all layers are used as a representation of the artistic style of the original image. The content and style are separate, and the data dimensions of the content and style are larger than the dimensions of the original image because they are expressed with several levels of abstraction. Using the same method, the gram matrices of the filter responses of all layers were extracted as a numerical representation of the style or feel of ride comfort. Extraction of the ride comfort did not require any modification of the networks. The only difference between the extraction of ride comfort and the extraction of the artistic style of an image was the nature of the input data. The input to the former was the measured temporal data, and the input to the latter was spatial data or an image. The temporal data were transformed into an image to use the pre-trained CNN without re-training the network.

#### 2.2.2. Preprocessing Input Data

A spectrogram of the temporal data was used to transform it into an image. Sometimes called a color map, a spectrogram is a visual representation of the spectrum of frequencies of a temporal signal as it varies with time. It effectively shows signal patterns in both the frequency domain and the time domain simultaneously. Most objective metrics for ride comfort are defined using characteristics in the frequency domain or in the time domain, which makes a spectrogram a good visual representation of the possible metrics of ride comfort.

**Figure 5:**
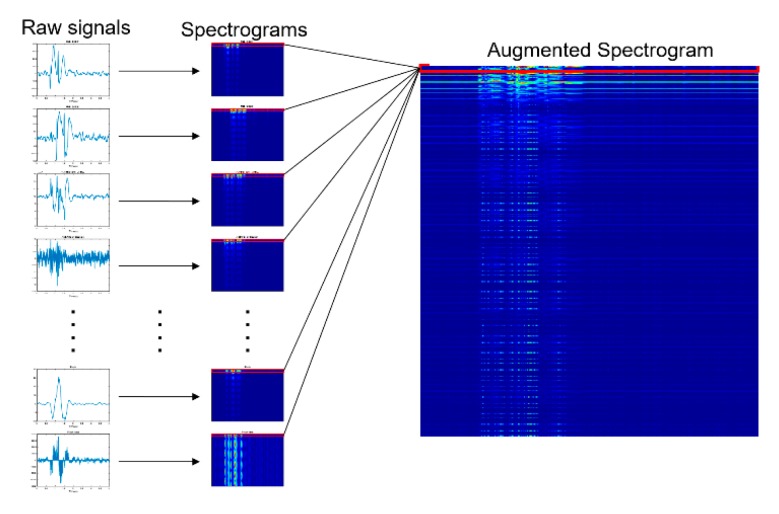
Preprocessing input data. Raw measured signals were transformed into an image for input.

Another issue in the transformation of the temporal data is that multiple spectrograms are generated from the temporal data because the data are a set of signals. A single image was input to the VGG-19 network, and thus multiple spectrogram images must be combined to feed them to the network. As shown in Figure 5, the spectrogram images were stacked line by line to strengthen the spectral and temporal correlations of the input data. Before the transformation, all signals were normalized so that the ranges of the values were between −1 and 1. Furthermore, the spectrograms were clipped along the frequency axis to remove the signal noise and bias.

#### 2.2.3. Vector of Ride Comfort Difference

**Figure 6:**
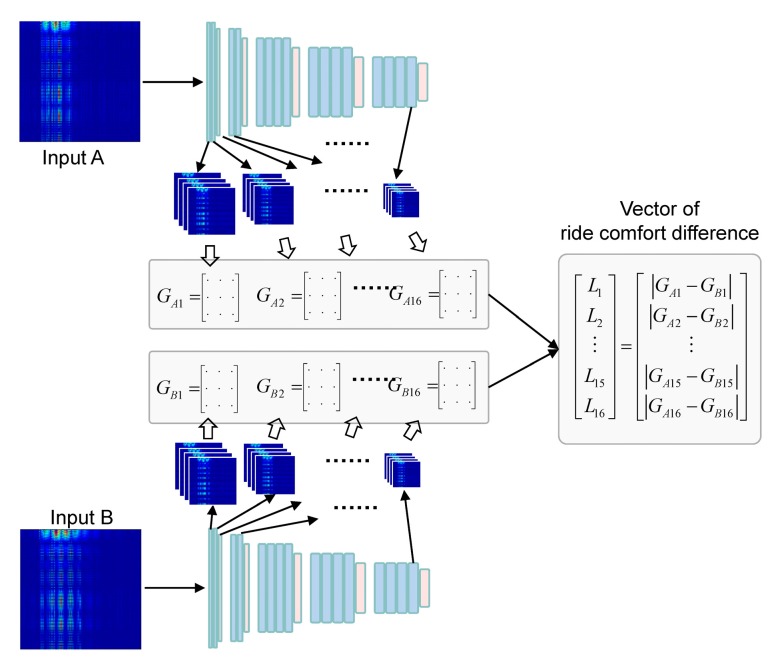
Comparative model of ride comfort. The output was a vector of the ride comfort difference. If the vector was a zero vector, the two vehicles had an identical ride comfort.

**Figure 7:**
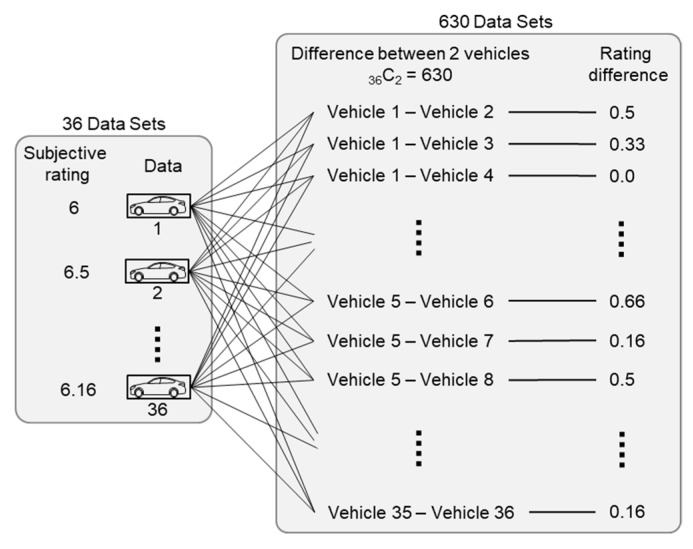
Expansion of datasets for model training.

Once the numerical representation of ride comfort was extracted, a neural network could be built to map from the numerical representation, [*G*_1_, *G*_2_, ···, *G*_16_], to the subjective rating. The output of the comparative model was the difference between the subjective ratings of two vehicles, and the input was the difference between the two numerical representations of the two vehicles. The set of norms of the gram matrix difference was defined as a vector of the ride comfort difference, as shown in Figure 6. This approach expanded the number of training sets from 36 to _36_C_2_ = 630, as shown in Figure 7.

#### 2.2.4. Comparative Model of Ride Comfort

**Figure 8:**
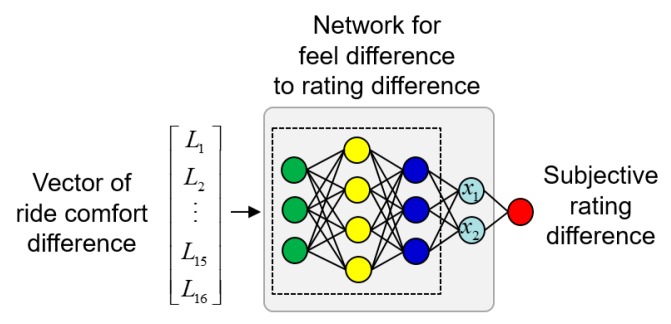
Correlation networks from the vector of ride comfort difference to the subjective rating difference. The neural network was composed of eight fully connected layers.

We designed a neural network for the correlation between the vector of ride comfort difference and the subjective rating difference. The network had eight fully connected hidden layers, as shown in Figure 8. The last hidden layer was designed for the visualization of the results with two nodes, *x*_1_ and *x*_2_.

## 3. Results and Discussion

For both the primary ride comfort and impact comfort, 500 out of 630 datasets were used for training, and 130 sets were used for testing. Seven measured signals were used as the input data. The signals were the front-wheel damping force, rear-wheel damping force, vertical acceleration of the center of gravity, vertical acceleration of the left seat rail, vertical acceleration of the right seat rail, pitch, and pitch rate.

For the primary ride comfort, the root mean square error (RMSE) for training was 0.0049, and that of the test was 0.0465. For impact comfort, the RMSE for training was 0.0040, and that of the test was 0.071. The RMSEs of previously reported correlation models for the objectification of subjective evaluation were in the range of 0.1 to 0.7 [13,17,20,27]. One possible reason for the higher accuracy of the proposed method was the use of a CNN that enabled rich feature extraction without the predefinition of features. Another reason was the use of a neural network for the correlation between the vector of ride comfort difference and the subjective rating difference.

**Figure 9:**
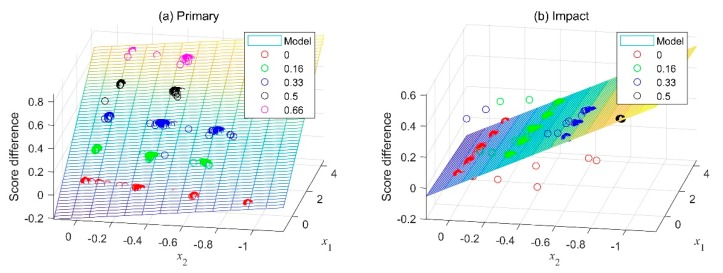
Three-dimensional view of the comparative models. The data points on the model were from test dataset. (**a**) Primary ride comfort (**b**) Impact ride comfort.

In Figure 9, the trained models for primary ride comfort and impact comfort were plotted as functions of *x*_1_ and *x*_2_, which were the node values of the last hidden layer. *x*_1_ predominantly affected the primary ride comfort, whereas *x*_2_ predominantly affected the ride comfort during impact. These models predicted how much the ride comfort differed between two given vehicles but did not give any information about which vehicle had the better ride comfort. One possible way to overcome this limitation was by comparing the ride comfort of a vehicle to that of another vehicle that had the lowest subjective rating.

**Figure 10:**
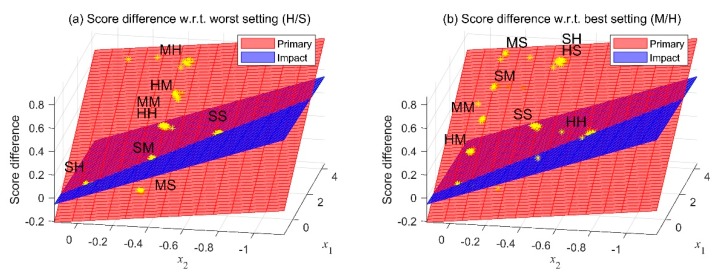
Comparative model (**a**) with respect to a vehicle with the lowest rating of ride comfort and (**b**) with respect to a vehicle with the highest rating of ride comfort.

In our data, the vehicle with the lowest rating was the one with the H/S damper setting. Figure 10a shows the result of the comparison to this vehicle. A result farther from the origin indicated a better ride comfort. A similar model could also be extracted using the vehicle with the highest subjective rating, as shown in Figure 10b. The vehicle with the highest ride comfort was the one with the M/H damper setting. In this case, a closer result to the origin meant a better ride comfort.

## 4. Case Study for Use of the Correlation Model

### Sensitivity Evaluation of Signal Changes to Ride Comfort

One useful application for this method is evaluating the sensitivity of the ride comfort rating to changes in the measured signals. The proposed model represented mapping functions from measured signals or vehicle dynamic states to subjective ratings. This model could tell what kinds of dynamic responses are beneficial for good ride comfort.

**Figure 11:**
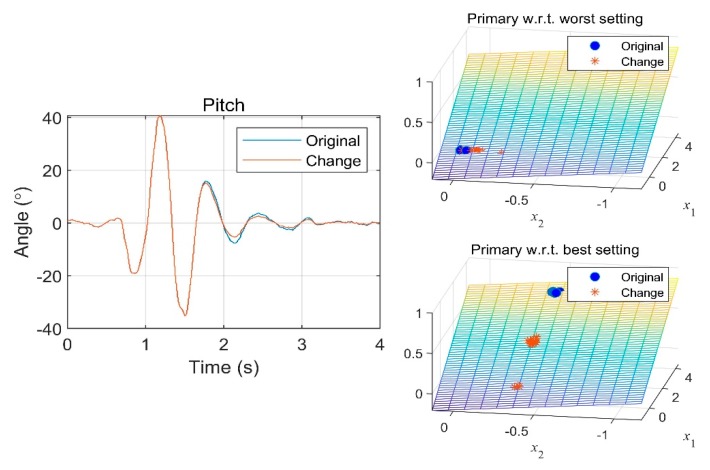
Ride comfort rating with respect to pitch decay rate change.

As examples of sensitivity analyses, we synthesized signals of the pitch angle and vertical acceleration, which were the most representative signals for primary ride comfort. Pitch angle variations that remained for a long time after passing over a speed bump negatively affected ride comfort. We synthesized the pitch angle signal such that it would decay faster after a speed bump. This signal change seemed to help improve primary ride comfort, as shown in Figure 11. The comparative model based on the worst-rated vehicle predicted a low correlation between this change and the ride comfort, but the comparative model based on the best-rated vehicle predicted a positive relationship. 

**Figure 12:**
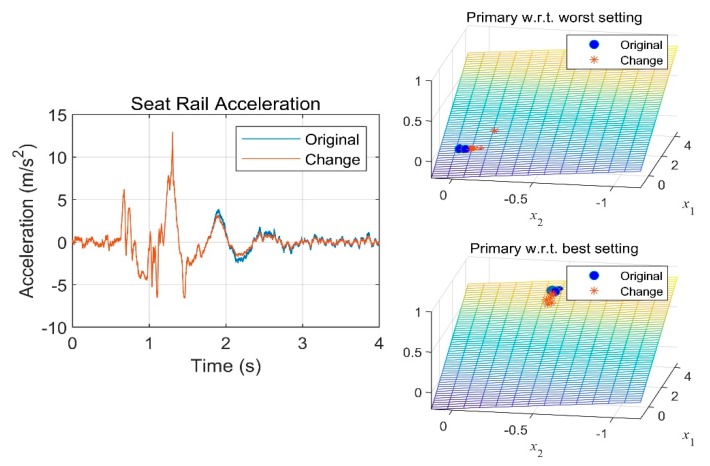
Ride comfort rating with respect to vertical acceleration change.

We performed a similar analysis with the vertical acceleration at the seat rail position. The vertical acceleration was synthesized to have a small magnitude reduction after passing over the bump, as shown in Figure 12. Both comparative models predicted no significant change in the primary ride comfort. 

**Figure 13:**
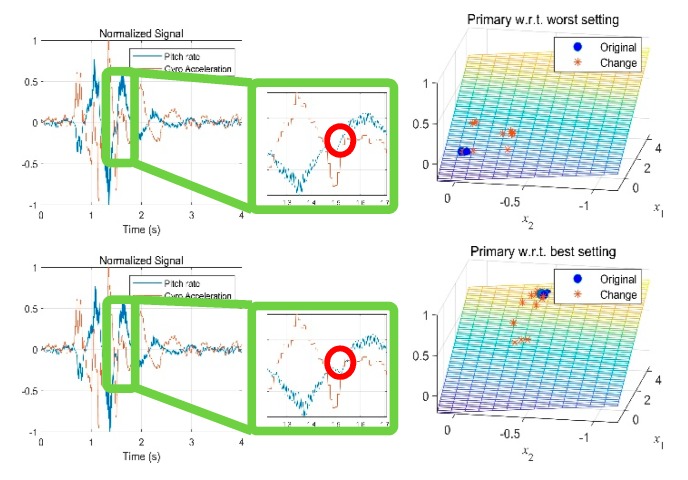
Ride comfort rating with respect to the change of phase difference between the pitch rate and vertical acceleration.

**Figure 14:**
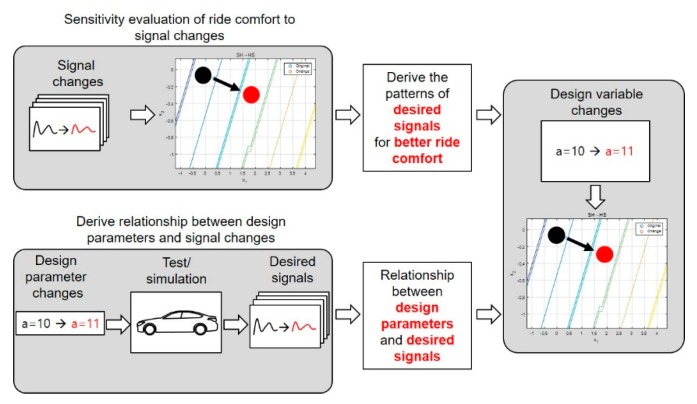
Possible use of evaluation model in vehicle design.

In the last case study, an analysis was performed on the reduction of the phase difference between the pitch rate and vertical acceleration at the center of gravity. When the phase difference was reduced, both comparative models predicted a significant improvement in the ride comfort, as shown in Figure 13. Other examples are shown in Table 3, which demonstrate that the proposed model could be used to identify the designed signal patterns without doing additional expensive experiments. Once the desired signal patterns were identified, tuning could be performed to improve the ride comfort in the early design stages if a simulation of a vehicle model with a high fidelity was available, as shown in Figure 14.

## 5. Conclusions

We have proposed a methodology to objectify subjective assessments using a pre-trained DNN. The proposed method does not require any feature definition or objective metric definition to extract ride comfort from measured signals. A DNN technique called artistic style transfer was used to extract a numerical form of the driving comfort without any predefined features, and then a comparative model was designed. The model showed a higher accuracy than any other correlation models in the literature. This was because of use of CNN in extracting performance metrics and use of a comparative model. The limitations of this research include a small number of test drivers, few test surfaces, and the limited number of datasets. These limitations can be a huge barrier to the use of a DNN, and thus, a general evaluator model could not be achieved with the given dataset. The limitations are typical and unavoidable for the subjective evaluation of vehicle ride comfort due to the expensive and long evaluation process. Therefore, the designed evaluator model does not work for all kinds of general conditions. Rather, the designed model can be used as a numerical evaluator model for the given road surface with the given vehicle speed. During the research, the authors concluded that designing a general evaluator model for vehicle subjective evaluation working for any conditions is practically impossible and designing several case-dependent models for each different test condition would be practically viable. The authors showed that the proposed method was effective for such cases. Even though the proposed method itself can be applied to both general and case-dependent models, the strength of the proposed method lies on the design of a subjective evaluation model when there is a small number of data points.

## Figures and Tables

**Table 1:** Subjective rating scale.

Rating Scale		Event Type	
		Disturbance	Control
10	Desirable	Imperceptible	Excellent
9		Trace	
8		A Little	Good
7		Some	
6		Moderate	Fair
5	Borderline		
4	Undesirable	Annoying	Poor
3		Strong	
2		Severe	Very Poor
1		Not Acceptable	

Most ride issues were disturbance events, while most handling issues were control events.

**Table 2:** Subjective evaluation results of ride comfort.

Damper Settings (Front/Rear)	Primary Ride	Impact (Secondary Ride)
H/H	6+	6
H/M	6+ to 7−	6+
H/S	6	6 to 6+
M/H	7−	6+
M/M	6+	6+
M/S	6	6+
S/H	6	6 to 6+
S/M	6 to 6+	6+
S/S	6+	6+ to 7−

H, M, and S stand for hard, medium, and soft, respectively.

**Table 3:** Subjective evaluation of the ride comfort in a speed bump test.

Cases	1	2	3	4	5	6	7
Pitch rate reduction	O	X	X	O	O	X	O
Acceleration reduction	X	O	X	O	X	O	O
Phase lag reduction	X	X	O	X	O	O	O
Improvement of primary ride comfort	Y	N	Y	Y	Y	Y	Y

## References

[1] Li C., Chen T. (2009). Aesthetic visual quality assessment of paintings. IEEE J. Sel. Top. Signal Process..

[2] Hayn-Leichsenring G.U., Lehmann T., Redies C. (2017). Subjective ratings of beauty and aesthetics: Correlations with statistical image properties in western oil paintings. I-Perception.

[3] Jeon J.Y., You J., Chang H.Y. (2007). Sound radiation and sound quality characteristics of refrigerator noise in real living environments. Appl. Acoust..

[4] Lee C., Cho Y., Baek B., Lee S., Hwang D., Jo K. Analyses of refrigerator noises. Proceedings of the IEEE International Symposium on Industrial Electronics.

[5] Xin Z., Zhang X., Shi G., Lin Y. Steering feel study on the performance of EPS. Proceedings of the IEEE Vehicle Power and Propulsion Conference.

[6] Huang F. G1 model of expressway ride comfort assessment. Proceedings of the International Conference on Measuring Technology and Mechatronics Automation.

[7] Zheng Y., Meng F., Zhang Y. A new subjective assessment system for video quality. Proceedings of the International Congress on Image and Signal Processing.

[8] Park H.-J., Har D.-H. (2011). Subjective image quality assessment based on objective image quality measurement factors. IEEE Trans. Consum. Electron..

[9] Wang E.M.-Y., Shih S.S.-Y. A study on thumb and index finger operated interface for personal mobile devices: Mobile phone keypad and joystick. Proceedings of the International Conference on Industrial Engineering and Engineering Management.

[10] Arima M., Tamura Y., Yoshihira M. Evaluation of ride comfort of passenger craft. Proceedings of the IEEE International Conference on Systems, Man and Cybernetics.

[11] Sawabe T., Okajima T., Kanbara M., Hagita N. Evaluating passenger characteristics for ride comfort in autonomous wheelchairs. Proceedings of the IEEE 20th International Conference on Intelligent Transportation Systems.

[12] Zschocke A.K., Albers A. (2008). Links between subjective and objective evaluations regarding the steering character of automobiles. Int. J. Automot. Technol..

[13] Chabrier E., Grima M. Subjective and objective vehicle tests, two parallel vehicle handling evaluations. Proceedings of the FISITA 2012 World Automotive Congress.

[14] Furusho Y., Kotani K. Objective and subjective evaluation models of pencil still drawings for art education. Proceedings of the International Conference on Digital Image Computing: Techniques and Applications.

[15] Data S., Frigerio F. (2002). Objective evaluation of handling quality. Proc. Inst. Mech. Eng. Part D J. Automob. Eng..

[16] Rothhämel M., Ijkema J., Drugge L. (2011). A method to find correlations between steering feel and vehicle handling properties using a moving base driving simulator. Veh. Syst. Dyn..

[17] Nybacka M., He X., Gil Gómez G.L., Bakker E., Drugge L. (2014). Links between subjective assessments and objective metrics for steering. Int. J. Automot. Technol..

[18] Nybacka M., He X., Su Z., Drugge L., Bakker E. (2014). Links between subjective assessments and objective metrics for steering, and evaluation of driver ratings. Veh. Syst. Dyn..

[19] Gil Gómez G.L., Nybacka M., Bakker E., Drugge L. (2015). Findings from subjective evaluations and driver ratings of vehicle dynamics: Steering and handling. Veh. Syst. Dyn..

[20] Gil Gómez G.L., Nybacka M., Bakker E., Drugge L. (2016). Objective metrics for vehicle handling and steering and their correlations with subjective assessments. Int. J. Automot. Technol..

[21] Chen G., Zhang W., Gong Z., Sun W. A new approach to vehicle shift quality subjective evaluation based on fuzzy logic and evidence theory. Proceedings of the IEEE Conference on Industrial Electronics and Applications.

[22] Liu Y., Liu Q., Lv C., Zheng M., Ji X. (2018). A Study on objective evaluation of vehicle steering comfort based on driver’s electromyogram and movement trajectory. IEEE Trans. Hum.-Mach. Syst..

[23] Varga D. (2019). No-Reference Video Quality Assessment Based on the Temporal Pooling of Deep Features. Neural Process. Lett..

[24] Mahendran N., Vincent D.R., Srinivasan K., Chang C.-Y., Garg A., Gao L., Reina D.G. (2019). Sensor-Assisted Weighted Average Ensemble Model for Detecting Major Depressive Disorder. Sensors.

[25] Weber K.A., Smith A.C., Wasielewski M., Eghtesad K., Upadhyayula P.A., Wintermark M., Hastie T.J., Parrish T.B., Mackey S., Elliott J.M. (2019). Deep Learning Convolutional Neural Networks for the Automatic Quantification of Muscle Fat Infiltration Following Whiplash Injury. Sci. Rep..

[26] Yao B.-G., Peng Y.-L., Yang Y.-J. (2018). Mechanical Measurement System and Precision Analysis for Tactile Property Evaluation of Porous Polymeric Materials. Polymers.

[27] Gil Gómez G.L., Nybacka M., Drugge L., Bakker E. (2018). Machine learning to classify and predict objective and subjective assessments of vehicle dynamics: The case of steering feel. Veh. Syst. Dyn..

[28] Mordvintsev A., Olah C., Tyka M. DeepDream-A Code Example for Visualizing Neural Networks. https://ai.googleblog.com/2015/07/deepdream-code-example-for-visualizing.html.

[29] Gatys L.A., Ecker A.S., Bethge M. Image style transfer using convolutional neural networks. Proceedings of the IEEE Conference on Computer Vision and Pattern Recognition.

[30] Simonyan K., Zisserman A. (2014). Very deep convolutional networks for large-scale image recognition. arXiv.

[31] (2016). SAE International Surface Vehicle Recommended Practice, Subjective Rating Scale for Vehicle Ride and Handling.

